# Balloon Enteroscopy‐assisted Endoscopic Retrograde Cholangiopancreatography for Choledocholithiasis in Situs Inversus Totalis With Billroth II Reconstruction: A Case Report and Literature Review

**DOI:** 10.1002/deo2.70403

**Published:** 2026-08-13

**Authors:** Shuhei Shintani, Shunya Oi, Kiyohiro Saito, Erina Yoshida, Noriaki Yamashita, Atsushi Nishida, Takuji Iwashita

**Affiliations:** ^1^ Department of Gastroenterology Shiga University of Medical Science Shiga Japan

**Keywords:** balloon enteroscopy, Billroth II reconstruction, choledocholithiasis, endoscopic retrograde cholangiopancreatography, situs inversus totalis

## Abstract

Endoscopic retrograde cholangiopancreatography (ERCP) in patients with situs inversus totalis (SIT) and Billroth II reconstruction is technically challenging because of mirror‐image anatomy, altered access to the papilla, and inverted papillary orientation. A 56‐year‐old man with a history of distal gastrectomy with Billroth II reconstruction for a gastric ulcer 20 years earlier was found to have common bile duct stones on pre‐ERCP imaging. ERCP was performed in the prone position, as in conventional ERCP, with the endoscopist standing on the patient's right side. A short‐type single‐balloon enteroscope was advanced through the afferent limb to the major papilla. Biliary cannulation was achieved using a standard ERCP catheter without positional modification or sphincterotome. Cholangiography confirmed small common bile duct stones. Endoscopic papillary balloon dilation with an 8‐mm balloon was performed, followed by stone extraction using a basket catheter. Final cholangiography confirmed complete stone clearance, and no adverse events, including post‐ERCP pancreatitis, occurred. This case suggests that favorable alignment between the 7 o'clock working‐channel direction and the bile duct axis may allow conventional‐position ERCP in selected patients with SIT and Billroth II reconstruction.

## 1 Introduction

Situs inversus totalis (SIT) is a rare congenital anomaly characterized by mirror‐image transposition of the thoracoabdominal organs [1, 2]. In endoscopic retrograde cholangiopancreatography (ERCP), differences in endoscopic and fluoroscopic orientation, access to the major papilla, and the direction of biliary cannulation require adjustments in spatial orientation and endoscopic manipulation [3]. Various modifications in patient positioning, endoscopist positioning, and procedural techniques have been proposed for ERCP in patients with SIT [4].

Surgically altered anatomy further complicates ERCP. In patients with Billroth II reconstruction, the papilla is accessed through the afferent limb, and cannulation is performed toward an inverted papilla. Therefore, in patients with both SIT and Billroth II reconstruction, clinicians must simultaneously address mirror‐image anatomy and postoperative gastrointestinal alterations.

Here, we report successful balloon enteroscopy‐assisted ERCP for choledocholithiasis in a patient with both SIT and Billroth II reconstruction.

## 2 Case Report

A 56‐year‐old man with a history of distal gastrectomy with Billroth II reconstruction for a gastric ulcer 20 years earlier presented with recurrent postprandial abdominal pain due to cholelithiasis and was scheduled for cholecystectomy. Preoperative evaluation revealed common bile duct stones, and endoscopic removal was indicated.

**FIGURE 1:**
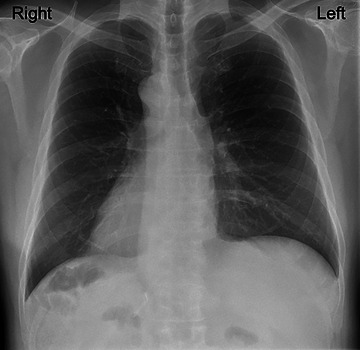
Chest radiograph demonstrating situs inversus totalis. Chest radiography demonstrated dextrocardia and a mirror‐image arrangement of the thoracoabdominal organs.

**FIGURE 2:**
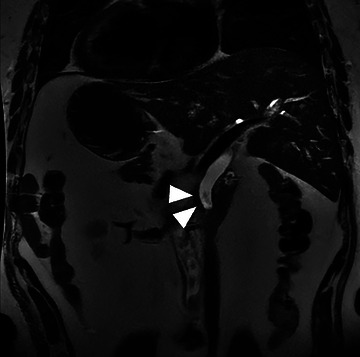
Magnetic resonance cholangiopancreatography demonstrating choledocholithiasis. Magnetic resonance cholangiopancreatography showed small filling defects in the distal common bile duct (arrowhead).

Laboratory findings showed no elevation of hepatobiliary enzymes, bilirubin, or other inflammatory markers. Chest radiography demonstrated mirror‐image thoracoabdominal anatomy, confirming SIT (Figure 1). Magnetic resonance cholangiopancreatography showed a small filling defect in the distal common bile duct (Figure 2). Endoscopic ultrasound thoroughly evaluated the entire common bile duct and identified several small stones, the largest measuring approximately 4 mm.

**FIGURE 3:**
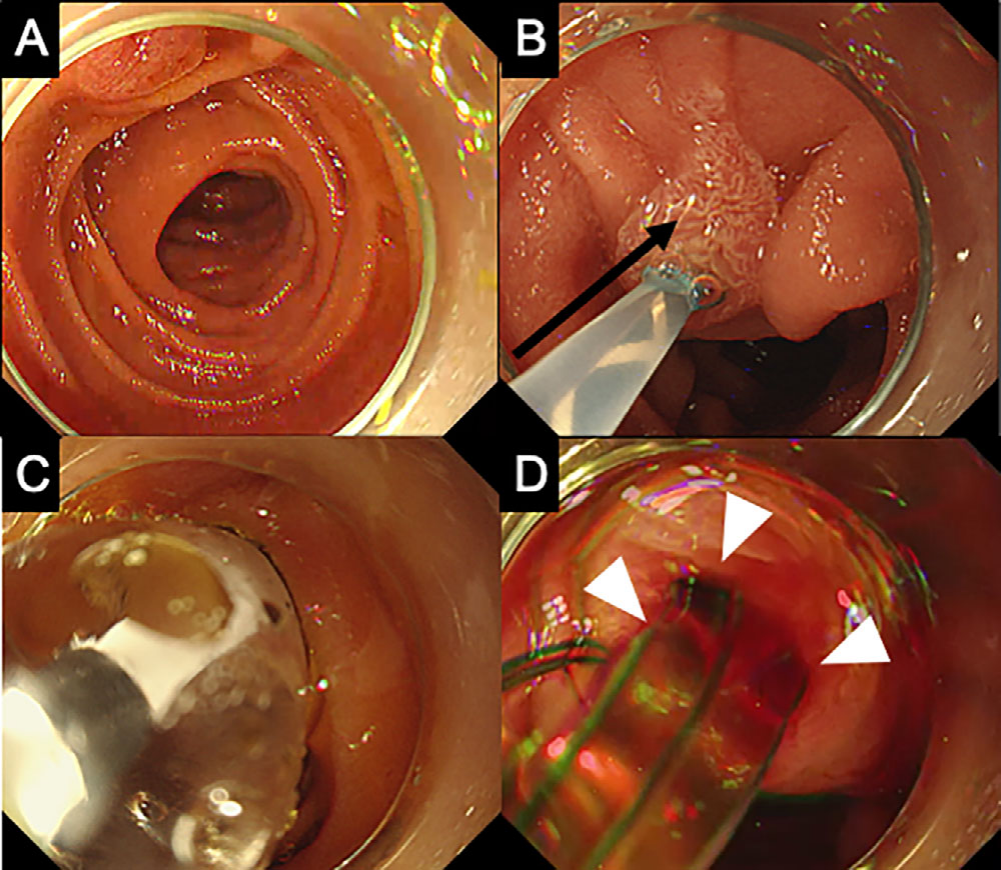
Balloon enteroscopy‐assisted endoscopic retrograde cholangiopancreatography. (A) Endoscopic view of the major papilla after advancement through the afferent limb using a short‐type single‐balloon enteroscope. (B) The papilla was located at the 10 o'clock position, and the device inserted through the 7 o'clock working channel aligns with the bile duct axis (arrow). (C) Endoscopic papillary balloon dilation using an 8‐mm dilation balloon. (D) Common bile duct stone extraction with a basket catheter. Arrowheads indicate the removed stones.

**FIGURE 4:**
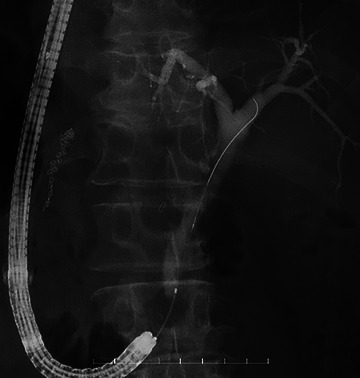
Fluoroscopic image during biliary cannulation.

ERCP was performed using a cap‐fitted short‐type single‐balloon enteroscope (SIF‐290S; Olympus, Tokyo, Japan) (Figure 3). The patient was placed in the prone position, and the endoscopist stood on the patient's right side, consistent with conventional ERCP. The enteroscope was advanced through the gastrojejunostomy into the afferent limb without difficulty, allowing visualization of the major papilla. Biliary cannulation was achieved using a standard ERCP catheter (MTW ERCP Catheter; ABIS, Hyogo, Japan). The catheter emerged from the 7 o'clock working‐channel direction and was advanced toward the papillary orifice. Cannulation was successful without the need for a sphincterotome. Selective biliary cannulation was achieved within 1 min after identification of the major papilla. Cholangiography confirmed small stones in the distal common bile duct (Figure 4). Endoscopic papillary balloon dilation (EPBD) was performed using an 8‐mm balloon (Hurricane; Boston Scientific, Marlborough, MA, USA), followed by stone extraction with a basket catheter (RASEN2; Kaneka Medix Corp., Osaka, Japan). Final cholangiography showed complete stone clearance. The total procedure time was 23 min. No adverse events, including post‐ERCP pancreatitis, were observed.

## 3 Discussion

This case demonstrates successful short‐type single‐balloon enteroscopy‐assisted ERCP for choledocholithiasis in a patient with SIT and Billroth II reconstruction. The procedure was completed in the conventional prone position with the endoscopist standing on the patient's right side, and biliary cannulation was achieved using a straight ERCP catheter alone. A short‐type single‐balloon enteroscope was selected because the length and course of the afferent limb can vary after Billroth II reconstruction, making access to the papilla with a conventional side‐viewing duodenoscope potentially difficult.

Only three cases of ERCP in patients with SIT and surgically altered anatomy have been reported (Table 1) [5, 6, 7]. Kim et al. described ERCP for an intact papilla in a patient with SIT and Billroth II reconstruction using a cap‐fitted forward‐viewing endoscope [5]. Inoue et al. used balloon enteroscopy, as in the present case, but for a hepaticojejunostomy anastomotic stricture, requiring different technical considerations from selective biliary cannulation through a naive papilla [6]. Therefore, the reconstruction type and whether the papilla is naive should be assessed before ERCP to plan an appropriate cannulation strategy.

**TABLE 1:** Endoscopic retrograde cholangiopancreatography (ERCP) in situs inversus with surgically altered anatomy.

Case	Age	Sex	Reconstruction	Indication	Endoscope	Patient/Endoscopist position	Cannulation technique	Use of sphincterotome	Procedure	Procedure time (min)	Technical success	AEs
Kim et al. [5]	82	Female	Billroth‐II	Choledocholithiasis	Forward‐viewing scope	N.E.	No special cannulation technique	No	EPBD	N.E.	Yes	—
Inoue et al. [6]	50`s	Female	Roux‐en‐Y	Drainage	EI‐580BT	Prone/right side	No special cannulation technique	No	Mechanical dilation	24	Yes	—
Tanisaka et al. [7]	95	Male	Billroth I	Choledocholithiasis	JF‐260	Prone/right side	Pancreatic guidewire‐assisted biliary cannulation	Yes	EST	N.E.	Yes	—
Present case	56	Male	Billroth II	Choledocholithiasis	SIF‐290S	Prone/right side	No special cannulation technique	No	EPBD	23	Yes	—

Abbreviations: EPBD: endoscopic papillary balloon dilation, EST: endoscopic sphincterotomy, N.E., not explicitly reported.

Various technical modifications have been proposed to address mirror‐image anatomy in SIT during ERCP, including changes in patient position, endoscopist position, and scope manipulation. The mirror‐image technique, in which the endoscopist stands on the patient's left side in the prone position, can create a familiar spatial orientation. Ding et al. reported that 11 of 14 patients with SIT underwent ERCP in modified positions, including the supine, left lateral, or right lateral position, achieving successful biliary cannulation in 85.7% of cases [3]. Other approaches, such as the twist method involving 180° rotation of the endoscope, have also been described [8]. While effective, these approaches may increase procedural complexity by deviating from standard ERCP practice. Notably, in previously reported cases of SIT with surgically altered anatomy, including Roux‐en‐Y and Billroth‐II reconstruction, ERCP was performed without changes in the patient or endoscopist positioning. Consistent with these reports, the present procedure was completed in the conventional prone position with the endoscopist standing on the patient's right side. Although the insertion route was influenced by mirror‐image anatomy, access to the papilla was achieved without major difficulty. These findings suggest that, in selected patients with SIT and an afferent limb, ERCP may be feasible without substantial modification of positioning.

Biliary cannulation in SIT is generally considered challenging due to altered endoscopic and fluoroscopic orientations. Several techniques such as the use of a sphincterotome to adjust the cannulation angle have been proposed to address this issue [9, 10]. In patients with SIT without surgically altered anatomy, Ding et al. reported difficult cannulation in 71.4% of cases [3]. In the report by Inoue et al., selective biliary cannulation was not required because the intervention was performed through a hepaticojejunostomy anastomosis. In contrast, Tanisaka et al. reported difficult biliary cannulation in a patient with Billroth I reconstruction and an intact naive papilla, owing to a bile duct axis oriented toward the 1 o'clock direction; this difficulty was overcome using pancreatic guidewire‐assisted cannulation. Although both SIT and Billroth II reconstruction generally increase the technical complexity of ERCP, their anatomical effects may have partially compensated for each other in the present case. Mirror‐image transposition associated with SIT and the inverted orientation of the native papilla after Billroth II reconstruction may have resulted in favorable alignment among the papillary orifice, the direction of the working channel, and the bile duct axis. Kim et al. reported successful biliary cannulation in a patient with SIT and Billroth II reconstruction with an intact naive papilla, an anatomical setting similar to that of the present case. Using a forward‐viewing endoscope, they achieved cannulation by advancing a straight catheter toward the papillary orifice from the 7 o'clock direction. Similarly, in the present case, biliary cannulation was achieved by directly advancing a straight ERCP catheter from the 7 o'clock working‐channel direction toward the papillary orifice. Although difficult biliary cannulation had been anticipated and a rotatable sphincterotome was prepared, it was not required. One possible explanation is that the mirror‐image anatomy associated with SIT and the inverted orientation of the papilla after Billroth II reconstruction created a favorable alignment between the direction of the straight catheter and the bile duct axis in this case. However, this favorable relationship should not be assumed in all patients with SIT and Billroth II reconstruction, because it may vary according to the configuration of the afferent limb, scope position and torque, papillary orientation, and individual bile duct anatomy. Therefore, the cannulation strategy should be individualized after assessment of these relationships at the papilla.

Previous reports showed that endoscopic sphincterotomy (EST) was performed in 12 of 14 patients with SIT (85.7%), whereas large balloon dilation was among the other therapeutic manipulations used in six of 14 patients (42.8%). Although post‐ERCP pancreatitis has not been reported in these case series, mild bleeding occurred in one case (7.1%) following EST; however, the limited number of cases precludes definitive conclusions [3]. In cases of SIT with Billroth II reconstruction, including the present case, EPBD has been selected, with no reported bleeding or pancreatitis [5].

In this patient with SIT and Billroth II reconstruction, advancing a straight ERCP catheter from the 7 o'clock working‐channel direction toward the papillary orifice resulted in favorable alignment with the bile duct axis and allowed ERCP to be completed in the conventional prone position.

## Author Contributions

**Shuhei Shintani**: conceptualization; literature review; manuscript drafting. **Shunya Oi**: manuscript revision; data acquisition. **Kiyohiro Saito**: manuscript revision; data acquisition. **Erina Yoshida**: manuscript revision; data acquisition. **Noriaki Yamashita**: manuscript revision; data acquisition. **Atsushi Nishida**: manuscript revision; supervision. **Takuji Iwashita**: manuscript revision; supervision.

## Conflicts of Interest

The authors declare no conflicts of interest.

## Funding

The authors have nothing to report.

## Ethics Statement

N/A
